# Transcriptome Analysis and Discovery of Genes Relevant to Development in *Bradysia odoriphaga* at Three Developmental Stages

**DOI:** 10.1371/journal.pone.0146812

**Published:** 2016-02-18

**Authors:** Huanhuan Gao, Yifan Zhai, Wenbo Wang, Hao Chen, Xianhong Zhou, Qianying Zhuang, Yi Yu, Rumei Li

**Affiliations:** 1 Institute of Plant Protection, Shandong Academy of Agricultural Sciences, Jinan, China; 2 Institute of Quality Standard & Testing Technology for Agri-products, Shandong Academy of Agricultural Sciences, Jinan, China; Institute of Vegetables and Flowers, Chinese Academy of Agricultural Sciences, CHINA

## Abstract

*Bradysia odoriphaga* (Diptera: Sciaridae) is the most important pest of Chinese chive (*Allium tuberosum*) in Asia; however, the molecular genetics are poorly understood. To explore the molecular biological mechanism of development, Illumina sequencing and *de novo* assembly were performed in the third-instar, fourth-instar, and pupal *B*. *odoriphaga*. The study resulted in 16.2 Gb of clean data and 47,578 unigenes (≥125bp) contained in 7,632,430contigs, 46.21% of which were annotated from non-redundant protein (NR), Gene Ontology (GO), Clusters of Orthologous Groups (COG), Eukaryotic Orthologous Groups (KOG), and Kyoto Encyclopedia of Genes and Genomes (KEGG) databases. It was found that 19.67% of unigenes matched the homologous species mainly, including *Aedes aegypti*, *Culex quinquefasciatus*, *Ceratitis capitata*, and *Anopheles gambiae*. According to differentially expressed gene (DEG) analysis, 143, 490, and 309 DEGs were annotated as involved in the developmental process in the GO database respectively, in the comparisons of third-instar and fourth-instar larvae, third-instar larvae and pupae, and fourth-instar larvae and pupae. Twenty-five genes were closely related to these processes, including developmental process, reproduction process, and reproductive organs development and programmed cell death (PCD). The information of unigenes assembled in *B*. *odoriphaga* through transcriptome and DEG analyses could provide a detailed genetic basis and regulated information for elaborating the developmental mechanism from the larval, pre-pupal to pupal stages of *B*. *odoriphaga*.

## Introduction

The family Sciaridae (Diptera: Sciaroidea) is composed of approximately 347 species in 32 genera in China [1]. Some species of this group are important vegetables pests. For example, *Bradysia odoriphaga*—(Diptera: Sciaridae) is the most important pest of Chinese chive (*Allium tuberosum* Rottler ex Sprengel) [2], which is grown over a vast geographic area from Asia through the Middle East and to Europe and North America [3,4]. During the entire developmental phase, the third-instar *B*. *odoriphaga* damages chives the most. Tissue rot and 30–80% yield losses can result from larvae feeding on root, bulbs, and even immature stem of chives, whereas adults cause minimal direct plant damage [5]. Mature larvae decrease their feeding amount and prepare to overwinter in the soil or in the residue of chives as pupae, which can protect against drought, enemies, and pesticides during their pupal stages. In addition to Chinese chive, this insect also has a wide distribution of hosts, including welsh onion, garlic, onion, lettuce, cucumber, Chinese cabbage, and mushroom[6–9]. In order to decrease the damage of *B*. *odoriphaga*, until now, chemical control has been the most effective method for the control of them [10]. However, the sustained dependence on insecticides has led to pesticide residue, soil pollution, and insecticide resistance. The integrated management of *B*. *odoriphaga* now has become an urgent problem.

Although ecological and physiological aspects of *B*. *odoriphaga* have been studied in recent years, molecular biological research about *B*. *odoriphaga* is still very limited. Fortunately, in recent years, next-generation high-throughput transcriptome sequencing techniques have improved dramatically the efficiency and speed of gene discovery in the life sciences [11,12], especially for organisms without a reference genome. Many studies using this technology have surveyed the complex transcriptomes of various insects, including *Drosophila melanogaster* [13], *Bombyx mori* [14], *Anopheles gambiae* and *A*. *aegypti* [15], *Maruca vitrata* [16], and *Spodoptera exigua* [17]. Moreover, transcriptome sequencing is also an efficient way to measure transcriptome composition, uncover the information of functional genes, and differentially express genes for the insects with different kinds, during different developmental stages, and in different developmental conditions [18–20].

The characterization of larval transcriptomes was studied by *de novo* sequencing, and, in *B*. *odoriphaga* [21], researchers found 408 unigenes related to insecticide resistance and metabolism in 16,829 assembled unigenes. However, the molecular regulatory mechanisms of *B*. *odoriphaga* remained unexplored, especially those related to developmental transition. Therefore, to understand the mechanism of the developmental process, it is essential to develop DEGs from *B*. *odoriphaga* at different developmental stages. In this study, the third-instar larvae, fourth-instar larvae, and pupal insects were selected as the research objects due to the close association between their damage to plants and their development. As the homologous species of *B*. *odoriphaga*, the genomic publication of Diptera insects, including *A*. *aegypti* [22] and *C*. *quinquefasciatus* [23], also could provide some reference information for gene discovery of *B*. *odoriphaga*. In this study, the annotated transcriptome sequences and gene expression profiles could provide useful information for the identification of genes involved in regulating the developmental process of *B*. *odoriphaga* at the third-instar, fourth-instar and pupal stages. The assembled and annotated unigenes in the transcriptome will help us to better explore the key regulated genes related to development and provide the molecular basis for the developmental process and functional analysis of these key genes.

## Materials and Methods

### Insect Material

*Bradysia odoriphaga* were collected from bulbs of Chinese chives in Jinan (117°00’E, 36°40’N), Shandong Province, China during April 2014. They were reared on bulbs of Chinese chives for 4–5 consecutive generations at 25± 0.3°C, 60% relative humidity, and 24: 0 L: D in a climatecontrolled chamber. Coming from the same generation, individuals of *B*. *odoriphaga* in three developmental stages (third -instar, fourth-instar, pupa) were collected successively and stored at -80°C. Due to two replicates in each development stage, six samples in total were used for transcriptomic analysis. The species are common agricultural pests and not included in the ‘‘List of Protected Animals in China”. No specific permissions were required as these fields are experimental plots that belong to the Shandong Academy of Agricultural Sciences, Jinan, Shandong in China.

### Rna Isolation and Quality Controls

Total RNA was extracted from 30 mg insects in each sample using TRI Reagent (Sigma Life Science, USA) according to the manufacturer’s protocol. The quality and quantity and integrity of each RNA sample was assessed using a Nanodrop spectrophotometer (Thermo Scientific, USA), Qubit 2.0 (Life Tech, USA), Aglient 2100 (Life Tech, USA). Only the RNA samples with 260: 230 ratio from 2.0 to 2.5, 260: 280 ratio from 1.9 to 2.1and an RNA integrity number (RIN) more than 8.0, were used for the analysis.

### Illumina Sequencing and *De Novo* Assembly

According to the Illumina manufacturer’s instructions, mRNA of each sample was gathered using oligo (dT) magnetic beads and fragmented into short sequences in the presence of fragmentation buffer. The cleaved mRNA was transcribed with random hexamers, and then second-strand cDNA synthesis was performed by adding dNTPs, RNase H and DNA polymerase I. After the purification of cDNA using AMPure XP (Beckman Coulter, USA) beads, endrepair and ligation of adaptors, the products were selected by AMPure XP beads and amplified by polymerase chain reaction (PCR) to create a cDNA library.

The cDNA library was sequenced on an Illumina sequencing platform (Hiseq 2500) (Illumina, USA). The raw reads from the images were generated using Solexa GA pipeline 1.6 sequencing by synthesis. After removal of low-quality reads containing primer/adaptor sequences and trimming of read lengths using SeqClean, high-quality reads considered as clean data with an identity value of 95% and a coverage length of 125 bp were assembled *de novo* using Trinity (http://trinityrnaseq.sourceforge.net/) software and clustered using the De Bruijn graph algorithm [24]. Ten the unigene sequence was then generated after unbinding the De Bruijn graph. According to the alignment with the sequences in the unigene library, the mapped reads in the clean data of each sample were applied for quality evaluation of the transcriptome sequencing library, such as randomness of mRNA fragmentation and saturation tests of sequencing data.

### Similarity Search and Functional Annotation

Unigene annotation of *B*. *odoriphaga* in three developmental stages was performed by searching the GenBank database with the BLASTX algorithm (http://www.ncbi.nlm.nih.gov/). NR (ftp://ftp.ncbi.nih.gov/blast/db/), GO (http://www.geneontology.org/), COG (http://www.ncbi.nlm.nih.gov/COG/), KOG (http://www.ncbi.nlm.nih.gov/COG/) and KEGG (http://www.genome.jp/kegg/) annotations of the unigenes were determined using BLAST software.

### Differentially expressed genes among *B*. *Odoriphaga* at three developmental stages

Reads sequenced from each sample of *B*. *odoriphaga* at different developmental stages were aligned with the unigene library by Bowtie (http://bowtie-bio.sourceforge.net/index.shtml). To obtain relative expression levels in each sample, fragments per kilobase of transcript per killion mapped reads (FPKM) in each sample were counted and combined with RSEM [25]. To ensure the reliability of differential expression of genes, the Pearson’s Correlation Coefficient (r) was deeded as evaluation indicator of correlation of two biological replicates [26]. The differential gene expression analysis was then performed using DESeq (http://www.bioconductor.org/packages/release/bioc/html/DESeq.html) for three compared groups (e.g., third vs fourth: the comparison of third-instar and fourth-instar *B*. *odoriphaga*; third vs pupa: the comparison of third-instar and pupal insects; and fourth vs pupa: the comparison of fourth-instar and pupal insects). The significance of differential gene expression was assessed using the Benjamini-Hochberg. A gene was considered to be differentially expressed when results from the above tests were all significant at a level of False Discovery Rate (FDR) <0.01 and a ratio of FPKM value of two compared goups (FC) ≥2. The GO, COG, KOG and KEGG annotations were performed using BLAST software for the differential expression of genes.

### Reverse transcription (RT-PCR) and Quantitative real-time PCR (qRT-PCR) validation

Total RNA was extracted from *B*. *odoriphaga* at three developmental stages as described above. Approximately 2μg of total RNA per sample were reverse-transcribed using a reverse transcription system (Promega, China). Forty-five genes were selected randomly in the common differential expression of genes among three comparisons of third-instar and fourth-instar larvae, third-instar larvae and pupa, and fourth-instar larvae and pupa for quantitative qRT-PCR validation. The RT-PCR and qRT-PCR were performed with the PrimeScript RT-PCR Kit and the SYBR Premix Ex Taq Kit (TaKaRa, China), repectively, according to the manufacturer’s protocol. The primers for RT-PCR was S1 Table. *β*-actin and *β*-tubulin were reference genes. Gene-specific primers, shown in S2 Table, were used for detecting the relative quantification of each gene. qRT-PCR expression levels were compared based on the means of three independent experimental repeats. Internal control genes (β-actin and *β*-tubulin) and a no template control (NTC) sample (nuclease free water) were included for normalization. A relative quantitative method (△△Ct) [27]was used to calculate relative expression level.

### Activity assay of enzymes

100mg *B*. *odoriphaga* collected at three developmental stages, respectively, was used to assay the activity of enzymes. There biological replicates were designed for this study. According to the NR annotation of 45 genes selected randomly in the common differential expression of genes among three comparisons, ten DEGs that could be expressed as eight functional enzymes were selected to validate the gene expression profiles. Pectate lyase (PEL) activity and glucose dehydrogenase (GLD) activity were assayed as described by Kovtunovych et al. [28] and Fender et al. [29], respectively. The activity of glutathione s-transferase (GST) was detected according to Glutathione S-Transferase Fluorescent Activity Kit (Enzo life science, USA). The assay of Carboxylesterase (CarE) activity referenced the methods of Zou et al. [30]. Alpha-Amylase Assay Kit (Abnova, USA), Lipase Activity Assay Kit III (Sigma, USA), Nitric Oxide Synthase Assay Kit (Antibodies-online, Germany), Fatty Acid Synthase assay Kit (Solarbio, China) were used to assayed the activity of alpha-amylase (AA), lipase (Lip), nitric oxide synthase (NOS) and fatty acid synthase (FAS).

## Results

### Illumina sequencing and *de novo* assembly

Illumina sequencing and *de novo* assembly were performed through merging all the sample of *B*. *odoriphaga* at three developmental stages (third-instar and fourth instar larval and pupal stages), resulting in the generation of 47,578 unigenes in total and a total length of 40,913,658 bp, with an N50 length of 1,517 bp and mean length of 859.93 bp. The corresponding information of contigs and transcripts is also shown in Table 1. The size distribution indicated that the lengths of the 12,772 unigenes were more than 1000 bp (S1 Fig). A total of 7,632,430 contigs were assembled and generated into 64,548,073 clean reads from all the libraries (Tables 1 and 2). The pupae library produced the most data (11,411,520 clean reads), while the fourth-instar larvae library produced the fewest clean reads (10,434,294).As a whole, all libraries exhibited good quality, with an average of 86.95% of clean reads with base call quality at Q30 and 92.12% at Q20. According to the alignments with the sequences in the unigene library, the statistics of mapped reads in each sample are shown in Table 2. The quality evaluation of the transcriptome sequencing library was then carried out according to the randomness of mRNA fragmentation and saturation test of sequencing data. The results revealed that the mapped reads of each sample were distributed in mRNA equally and the randomness of mRNA fragmentation was good (S2 Fig). The sufficient and effective information was applied in this study due to the saturation of gene number with the increase of sequenced reads (S3 Fig). Therefore, the eligible transcriptome sequencing library could ensure the reliability of transcriptome sequencing results.

**Table 1:** Statistics for assembled unigenes.

	Contig	Transcript	Unigene
Total number	7,632,430	73,613	47,578
Total length	324,952,807	79,583,767	40,913,658
N50 length	44	1,885	1,517
Mean length	42.58	1081.11	859.93

**Table 2:** Aligning statistics of clean reads with assembled unigenes.

Sample	Clean reads	Clean nucleotides (bp)	> Q20%	> Q30%	(G + C) %	Mapped reads	Mapped ratio
3rd	10,777,559	2,714,438,439	92.37	86.77	42.25	8,999,053	83.47%
4th	10,650,135	2,681,557,388	91.02	86.30	43.79	8,507,621	79.91%
Pupa	10,846,343	2,732,196,321	92.96	87.78	39.94	9,301,375	85.76%
All	33,105,328	8,128,192,148					
Mean			92.12	86.95	41.99		

‘3rd’, ‘4th’ and ‘Pupa’ indicate two biological replicates of *Bradysia odoriphaga* at third-instar, fourth-instar and pupal stages respectively; Mapped ratio means the ratio of mapped reads in clean reads.

### Functional annotation of all unigenes

**Fig 1:**
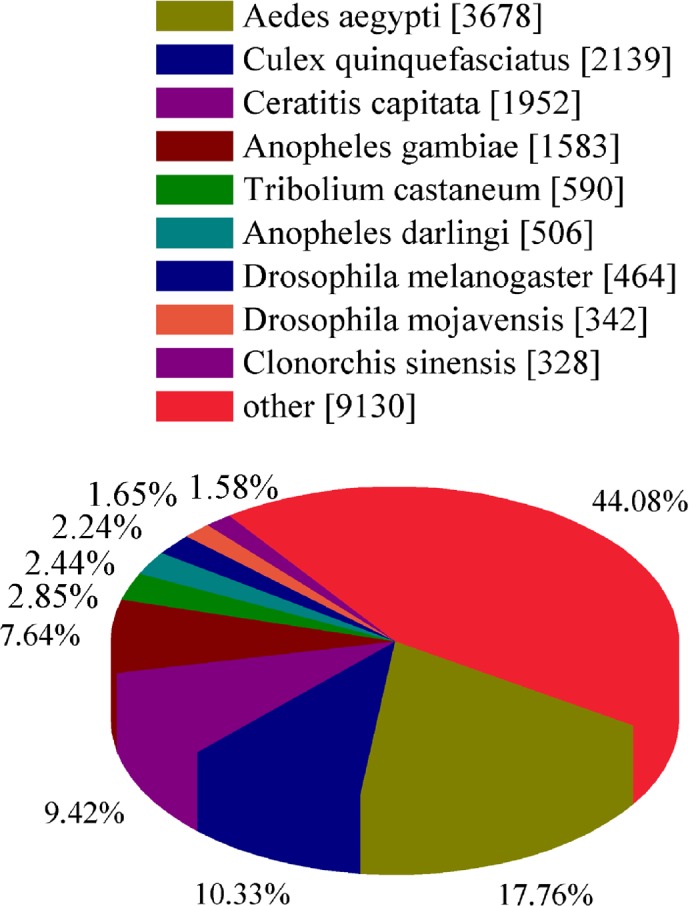
Match result of non-redundant protein (NR) homologous species distribution. The number means the matched unigenes with the corresponding homologous species for all unigenes of *Bradysia odoriphaga* at three developmental stages.

**Fig 2:**
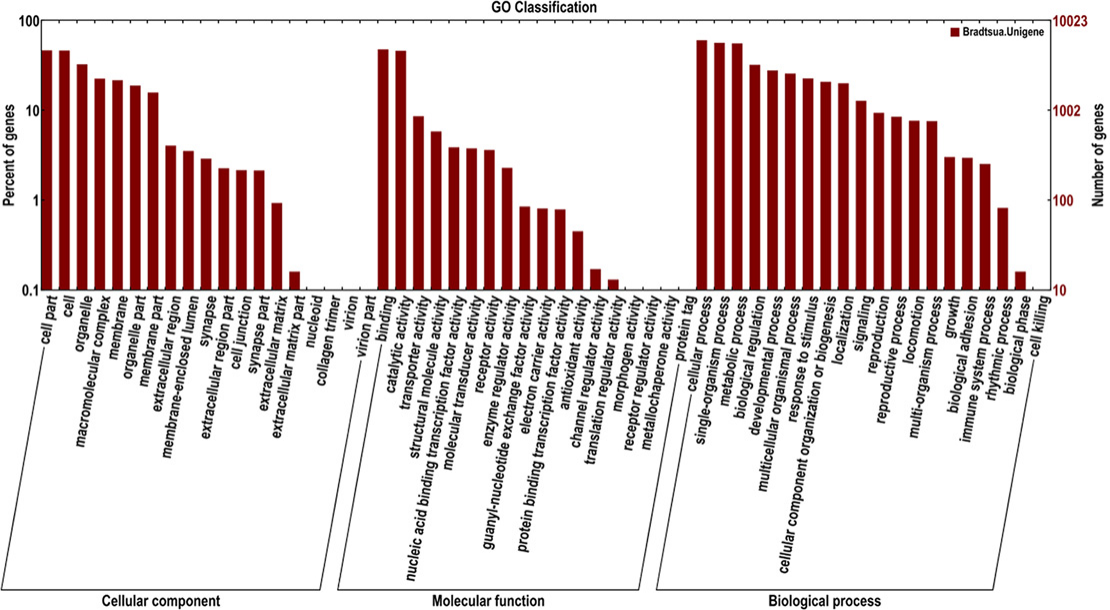
Number of *Bradysia odoriphaga* unigenes in each functional category. Unigenes were classified into different functional groups based on the Gene Ontology (GO) data library within cellular component, molecular function and biological process categories.

**Fig 3:**
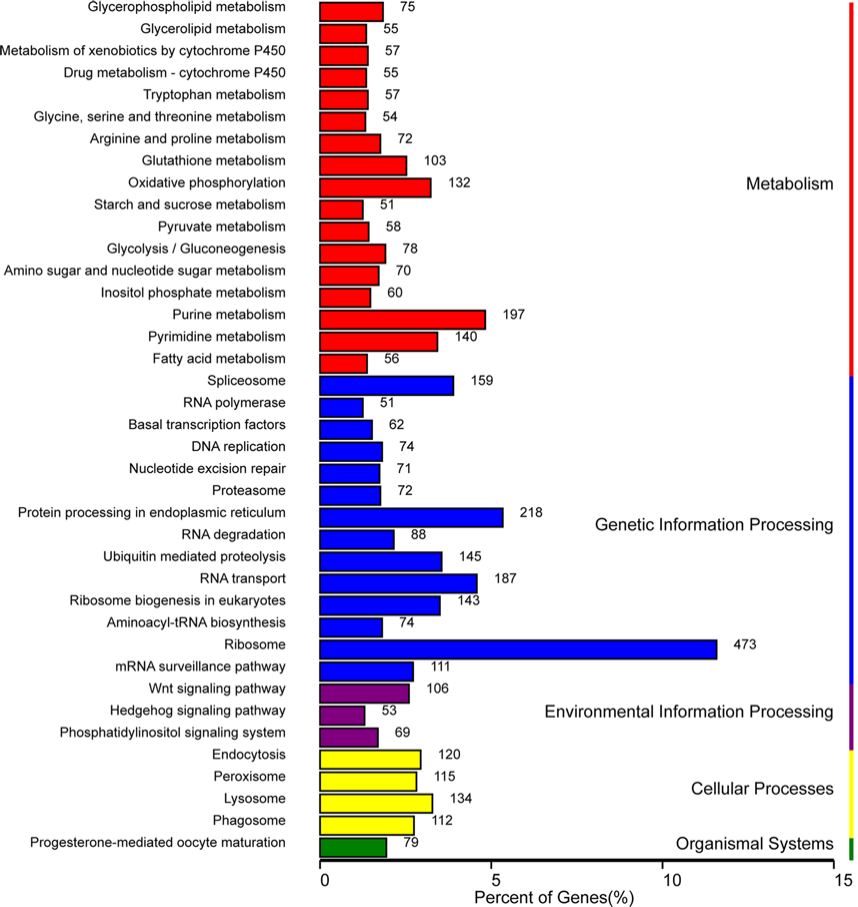
The distribution of pathways of differential expression genes (DEGs) annotated in the Kyoto Encyclopedia of Genes and Genomes (KEGG) data library.

To classify the functions of the predicted unigenes of three developmental stages, among 47,578 unigenes of *B*. *odoriphaga*, 21,985 of them were annotated from NR (21,095), GO (10,023), COG (7,856), KOG (14,712) and KEGG (6,820) database using a cut-off E-value of 10^−5^. For the NR annotation, 44.33% of all unigenes (47,578) provided a BLAST result, and the best-match results of NR homologous species distribution are shown in Fig 1. The sequences of *B*. *odoriphaga* showed 3,687 matches with *A aegypti* sequences followed by *C quinquefasciatus* (2,139), *C capitata* (1,952), and *A gambiae* (1,583). In each of the three main categories (cellular component, molecular function and biological process) of the GO classification, the terms “cell part”, “binding”, and “cellular process” were the most dominant, respectively (Fig 2). There are 2,794 genes related to the developmental process in the biological process category. In the KOG and COG functional classifications, “translation, ribosomal structure, and biologenesis” was the dominant function except for the general function predication only (S4 and S5 Figs). Five main categories (Metabolism, Cellular processes, Genetic information processing, Organism systems, Environmental information processing) in KEGG annotation are shown in Fig 3. Almost half of all unigenes (47.19%) were annotated as pathways related to the category “Genetic information processing”.

### Differentially expressed genes

According to the gene expression level (FPKM), the reliability of DEGs was analyzed by evaluating the correlation of two biological replicates in each developmental stage (S6 Fig). It was revealed that the gene expression tendency of two biological replicates was similar because the majority of points were clustered around the diagonal line.

**Fig 4:**
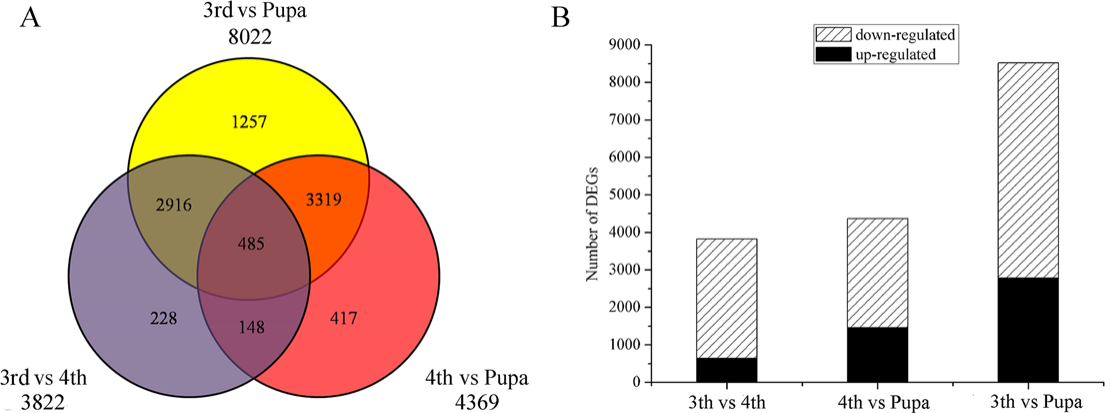
Information of differentially expressed unigenes in each comparison of *Bradysia odoriphaga*. (A) Comparison of differentially expressed unigenes in each comparison. (B) Regulated characteristics of differentially expressed unigenes in each comparison. Three comparisons of third-instar and fourth-instar *B*. *odoriphaga* (3rd vs 4th), third-instar and pupal insects (3rd vs Pupa), and fourth-instar and pupal insects (4th vs Pupa) are shown.

Based on the DEG analysis, among 3,822 genes expressed differentially between third and fourth-instar larvae, 643 and 3,197 genes were up-regulated and down-regulated, respectively. A total of 4,369 genes with significantly different expression levels was revealed between fourth-instar larvae and pupae. Among them, 1,462 and 2,907 genes were up-regulated and down-regulated, respectively, in the comparison of pupal stage and fourth-instar larvae. However, 8,022 genes expressed differentially were analyzed between third-instar and pupal *B*. *odoriphaga*; differential expression was most significant due to a longer interval of developmental stages (Fig 4A). In all the DEGs, with an increasing developmental stage, the number of down-regulated genes was more than that of up-regulated genes (Fig 4B). Moreover, the results revealed 485 DEGs all expressed differentially in three comparisons of third-instar and forth instar larvae, third-instar larvae and pupae, and fourth-instar larvae and pupae, some of which were selected and used for validation via qRT-PCR.

### Expression variation of DEGs related to the developmental process among three different developmental stages

To discover the genes related to the developmental process of *B*. *odoriphaga*, the functions of DEGs were annotated from GO, COG, KOG, and KEGG databases according to the annotated information of all unigenes of *B*. *odoriphaga*.

**Fig 5:**
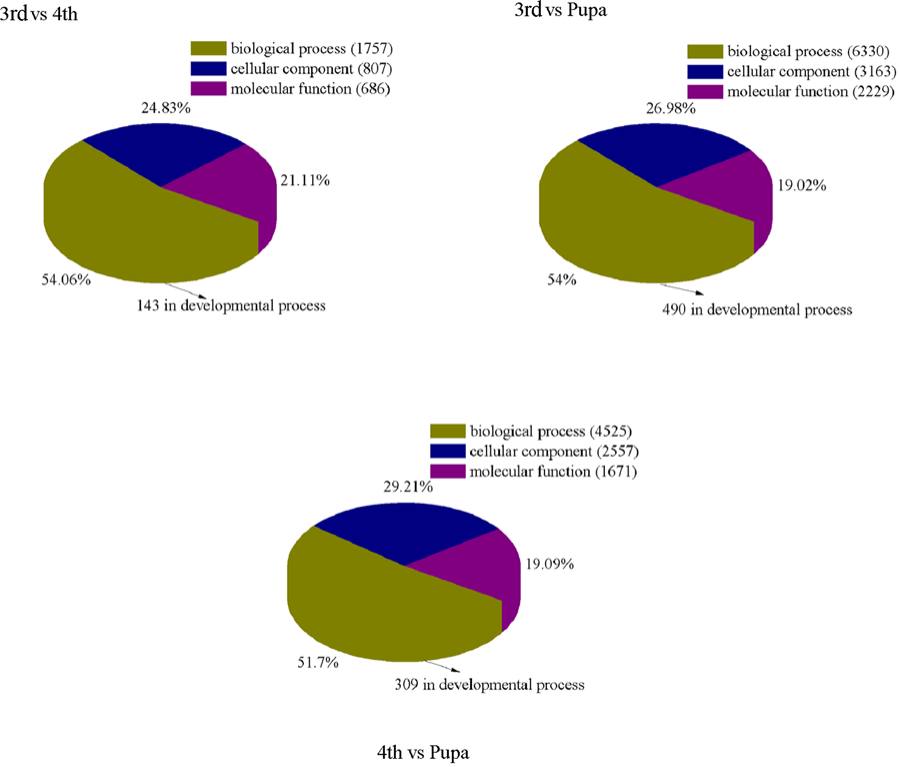
Numbers of differentially expressed genes (DEGs) annotated in the Gene Ontology (GO) data library in three comparisons. The results of genes related to the developmental process are shown. 3rd vs 4th: the comparison of third-instar and fourth-instar *Bradysia odoriphaga*; 3rd vs Pupa: the comparison of third-instar and pupal insects; 4th vs Pupa: the comparison of fourth-instar and pupal insects.

In the comparison of third-instar and fourth-instar larvae, 3,250 DEGs were annotated in the GO database (Fig 5). Among 143 genes related to the developmental process, three genes were selected according to the standard (log_2_FC≥2) (S3 Table). Genes *GL1336* and *HIV-1 rev binding protein 2 in Homo sapiens* were down-regulated in fourth-instar larvae compared to those of the third-instar stage. They were annotated as involved in the developmental process in the GO database (Tables 3 and 4), and the gene *GL1336* was involved in transcription according to the KOG and COG annotations (S4 Table). Expression of the gene *caspase Nc-like* was up-regulated in fourth-instar larvae compared to that in the third- instar stage. It positively regulated cell division and chromosome partitioning of the apoptotic process, tissue development, eye development and nervours system development (Tables 3 and 4, and S4 Table).

**Table 3:** Gene Ontology (GO) annotation of differentially expressed genes (DEGs) related to *Bradysia odoriphaga* development.

Unigene ID	NR annotation	GO annotation
c4843	*GL13336*	2
c23897	*HIV-1 rev binding protein 2 in Homo sapiens*	2
c33601	*caspase Nc-like*	8,9,19,24
c10244	*protein yellow-like*	3
c20752	*putative hunchback*	2
c23801	*tyrosine-protein kinase receptor torso*	1,4,9,10,11,16,25,26
c26961	*ecdysone-induced protein 63E*, *isoform N*	13,23
c27681	*protein ovarian tumor locus-like isoform X2*	1
c28314	*GG21746*	1,4,6,7,12,15,21,27
c28746	*dopamine N-acetyltransferase-like*	3
c30336	*conserved hypothetical protein*	3,5,6
c30501	*GH23593*	1,4
c30850	*fhos*, *isoform F*	5,17
c31327	*retinoblastoma family protein-like isoform X1*	1,7,8
c4206	*dynamin*	1,7,10,11,14
c15127	*hypothetical protein AaeL_AAEL011875*	2
c22888	*hidAla2*	5,13,18,20
c22990	*GG17085*	3
c24932	*GK10804*	12,14,21,22,28,29,30,31
c29289	*protein Dom3Z-like*	2,4,6
**C11110**	***PROBABLE H/ACA RIBONUCLEOPROTEIN COMPLEX SUBUNIT 1-LIKE***	**32**
**C28873**	***CYTOCHROME P450*, *PUTATIVE***	**33**
**C29314**	***NITRIC OXIDE SYNTHASE (NOS)***	**34**
**C16124**	***ACIDIC ENDOCHITINASE SP2-LIKE***	**35**
**C20905**	***GLUCOSE DEHYDROGENASE***	**36**

The bold content indicates the DEGs related to the developmental process in the common DEGs among three compared groups (3rd vs 4th, 4th vs Pupa, 3rd vs Pupa).

**Table 4:** Gene Ontology (GO) annotation of DEGs related with *Bradysia odoriphaga* development.

GO annotation	GO ID	Number
developmental process involved in reproduction	(GO:0003006)	1
developmental process	(GO:0032502)	2
developmental pigmentation	(GO:0048066)	3
cellular process involved in reproduction in multicellular organism	(GO:0022412)	4
developmental programmed cell death	(GO:0010623)	5
oogenesis	(GO:0048477)	6
cell development	(GO:0048468)	7
apoptotic process	(GO:0006915)	8
tissue development	(GO:0009888)	9
gamete generation	(GO:0007276)	10
organ development	(GO:0048513)	11
compound eye morphogenesis	(GO:0001745)	12
embryonic development via the syncytial blastoderm	(GO:0001700)	13
open tracheal system development	(GO:0007424)	14
embryo development	(GO:0009790)	15
embryonic morphogenesis	(GO:0048598)	16
salivary gland histolysis	(GO:0035070)	17
salivary gland cell autophagic cell death	(GO:0035071)	18
eye development	(GO:0001654)	19
eye pigment biosynthetic process	(GO:0006726)	20
imaginal disc-derived wing morphogenesis	(GO:0007476)	21
wing disc morphogenesis	(GO:0007472)	22
instar larval development	(GO:0002168)	23
nervous system development	(GO:0007399)	24
cell morphogenesis involved in neuron differentiation	(GO:0048667)	25
neuron projection morphogenesis	(GO:0048812)	26
neuron differentiation	(GO:0030182)	27
heart development	(GO:0007507)	28
stem cell development	(GO:0048864)	29
morphogenesis of embryonic epithelium	(GO:0016331)	30
cuticle pattern formation	(GO:0035017)	31
**CHITIN-BASED CUTICLE DEVELOPMENT**	**(GO:0040003)**	**32**
**TUBE DEVELOPMENT**	**(GO:0035295)**	**33**
**REGULATION OF ORGAN GROWTH**	**(GO:0046620)**	**34**
**MITOTIC SPINDLE ELONGATION**	**(GO:0000022)**	**35**
**MESODERM DEVELOPMENT**	**(GO:0007498)**	**36**

The bold content indicates the DEGs related with developmental process in the common differential expression genes among three compared groups (3rd vs 4th, 4th vs Pupa, 3rd vs Pupa).

The comparison between fourth-instar and pupal *B*. *odoriphaga* revealed 309 DEGs related to the developmental process (Fig 5). Among them, 11 genes were selected according to the standard (log_2_ FC≥2) (S3 Table) and all were up-regulated in the pupal stage compared to the fourth-instar larvae. They were participated in the “developmental process involved in reproduction”, “developmental process”, “developmental pigmentation”, “developmental programmed cell death” and reproductive organs development such as oogenesis, gamete and embry (Tables 3 and 4). In the KOG annotation, a gene encoding a tyrosine-protein kinase receptor regulated signal transduction mechanism through mitogen-activated protein kinase (MAPK) signaling pathway (S4 Table). Gene *GG21746* positively regulated the process of imaginal disc-derived wing morphogenesis. Moreover, *ecdysone-induced protein 63E* not only regulated instar larval development but also was involved in embryonic development in the pupal stage (Tables 3 and 4).

In the comparison of third-instar and pupal insects, although 19 genes were selected among 490 DEGs (Fig 5) related to the developmental process according to the standard (log_2_ FC≥2) (S3 Table), the majority of them showed similarly regulated profile and function. Similar to the comparison of fourth-instar and pupal stages, 11 genes positively regulated the developmental processes of reproduction, PCD, and reproductive organs in the pupal stage compared to those of the third-instar larvae. With the exception of those, the expression levels of genes *dynamin* and *GK10804* related to the proceses of the open tracheal system, heart, and stem cell development weres lower in the pupal stage of *B*. *odoriphaga*.

Moreover, among 45 genes selected of the 485 common DEGs among three comparisons of third-instar and fourth-instar larvae, third-instar larvae and pupae, and fourth-instar larvae and pupae, five DEGs was related to the developmental process, such as chitin-based cuticle development (*probable H/ACA ribonucleoprotein complex subunit 1-like*), tube development (*P450*), regulation of organ growth (*NOS*), mitotic spindle elongation (*acidic endochitinase SP2-like*) and mesoderm development (*glucose dehydrogenase*) (Table 3). According to the functional annotation, it was found that, nitric oxide synthase (NOS) regulated the organ growth and energy production and conversion negatively through regulating the “arginine and proline metabolism” pathway.

### Reverse transcription (RT-PCR) and Quantitative real-time PCR validation

**Fig 6:**
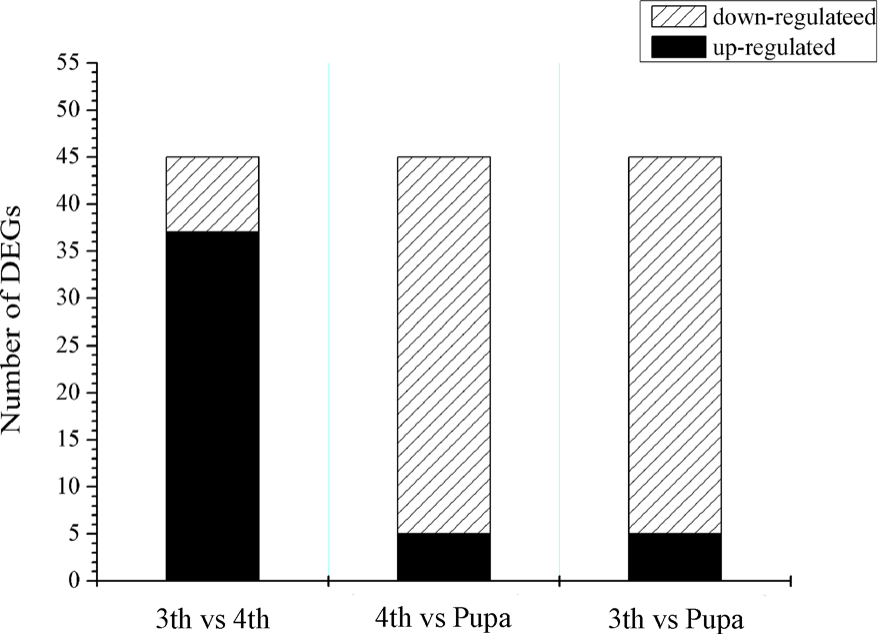
Numbers of 45 differentially expressed genes (DEGs) together in each comparison. Three comparisons of third-instar and fourth-instar *Bradysia odoriphaga* (3rd vs 4th), third-instar and pupal insects (3rd vs Pupa), and fourth-instar and pupal insects (4th vs Pupa) are shown.

Forty-five genes selected among 485common DEGs in three comparisons of third-instar and fourth-instar larvae, third-instar larvae and pupae, and fourth-instar larvae and pupae were classified into four gene expression profiles, such as, those up-regulated in fourth-instar and pupal stages compared to third-instar stage (I); down-regulated in fourth-instar and pupal stages compared with third-instar stage (II); down-regulated in fourth-instar and up-regulated in pupal stages compared to third-instar stage (III); up-regulated in fourth-instar and down-regulated in pupal stage compared to third-instar stage (IV). There were two, five, three, and 35 genes in the four gene expression profiles respectively (S5 Table and Fig 6).

**Fig 7:**
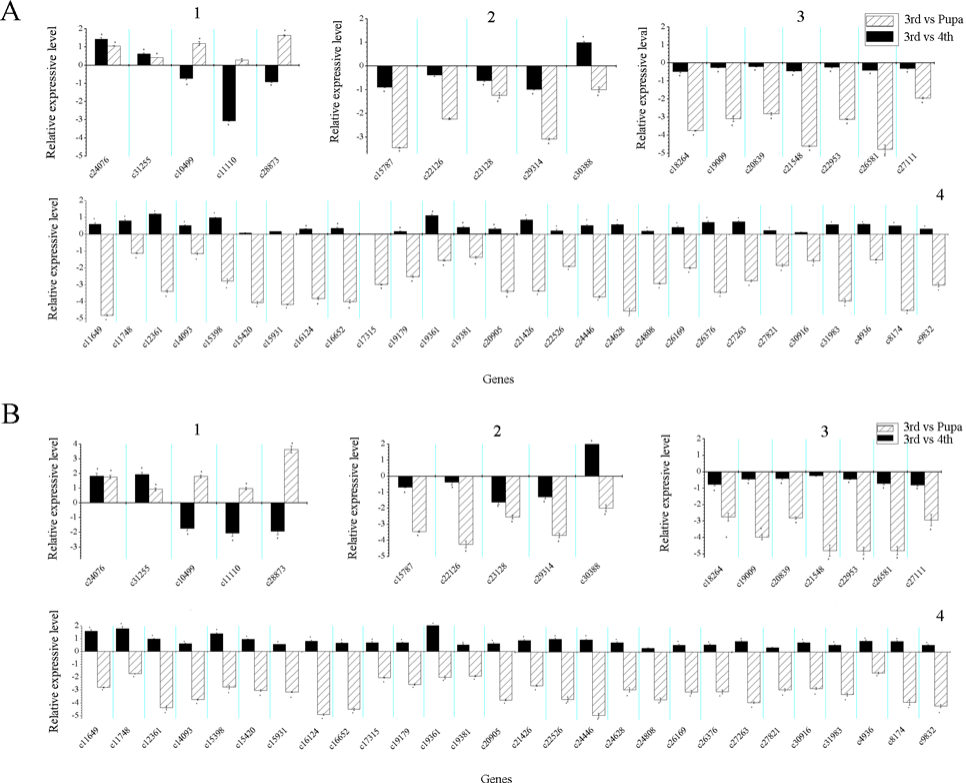
Quantitative real-time polymerase chain reaction (qRT-PCR) analysis of gene expression in fourth-instar and pupal-stage *Bradysia odoriphaga*. (A) *β*-actin was a reference gene. (B) *β*-tubulin was a reference gene. (1) The genes c24076 and c31255 belong to regulated type I, while c10499, c11110, and c28873 belong to type III. (2) qRT-PCR analysis of type II gene expression; (3) qRT-PCR analysis of inconsistent type IV gene expression. (4) qRT-PCR analysis of type IV gene expression. qRT-PCR data calculated with the 2^-△△Ct^ method. The value of relative expression level on the y-axis calculated according to log(2^-△△Ct^). The expression level of each gene in third-instar insects was arbitrarily set as 1, the value of the y-axis was 0, and its corresponding transcript levels in fourth-instar (3rd vs 4th) and pupal stages (3rd vs Pupa) were calibrated against the third one. *, p < 0.05, as determined by one-way analysis of variance (ANOVA).

To further validate the gene expression profiles, RT-PCR and qRT-PCR were carried out for the 45 DEGs among the three compared groups. The number of genes in each gene expression profiles through qRT-PCR (Fig 7) were almost invariant compared with the electronic data of gene expression analysis (S7 Fig). The expression profiles of genes analyzed by two methods, such as qRT-PCR and RT-PCR (S8 Fig), were consistent completely. It was revealed that the gene expression profiles were similar under the condition of two reference genes (A: *β*-actin; B: *β*-tubulin). The majority of genes were up-regulated in fourth-instar and down-regulated in pupal stage compared to those of the third-instar stage. For example, these included metabolism-related genes (*lipase*, *delta-5 desaturase*, *acidic endochitinase*, *pectate lyase*, *lysosomal alpha-mannosidase*, *regucalcin*, etc.), defense response related genes (*cytochrome P450*, *glutathione S-transferase*, *carboxylesterase*, *defensin*, etc.) (S5 Table). qRT-PCR results showed that, two type I genes, three type III genes (Fig 7A-1 and 7B-1) and 28 type IV genes (Fig 7A-4 and 7B-4) were confirmed from the results of the DEG analysis. Only gene c30388 of type II (Fig 7A-2 and 7B-2) and seven genes in type IV (Fig 7A-3 and 7B-3) were not confirmed from the results of the DEG analysis. Therefore, statistically, 82.22% of genes in the qRT-PCR results were in accordance with the electronic data of gene expression analysis.

For the five DEGs related to the development process, their qRT-PCR results were identical to those obtained by DEG expression profiling. Genes *probable H/ACA ribonucleoprotein complex subunit 1-like* (c11110) related to chitin-based cuticle development and *P450* (c28873) related to tube development were down-regulated in fourth-instar-stage and up-regulated in pupal stage compared to third- instar insects (Fig 7A-1 and 7B-1). Similar to the digital analysis results, *NOS* (c29314) related to organ growth, was negatively regulated in fourth-instar and pupal-stage compared to the third- instar *B*. *odoriphaga* (Fig 7-2).

### Activity assay of enzymes

**Fig 8:**
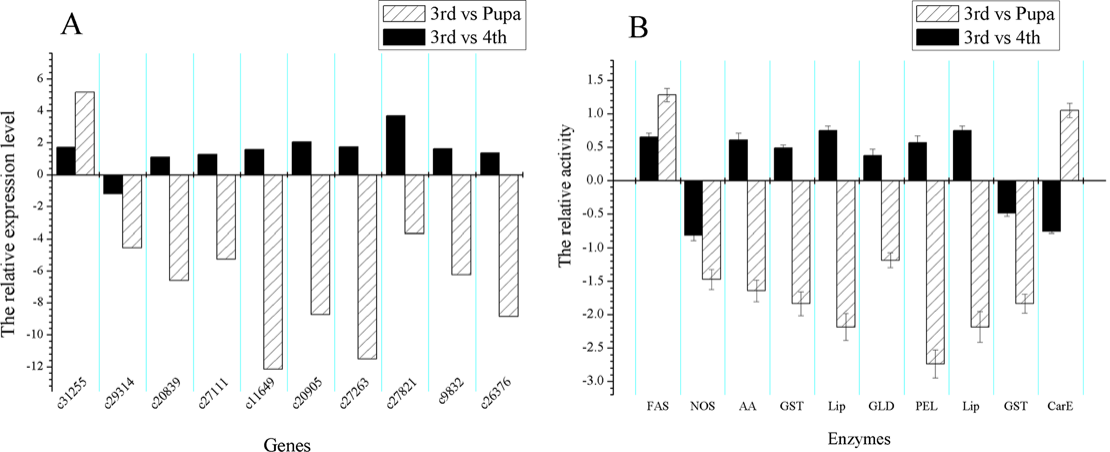
The relative gene expression level of ten DEGs and relative activity of functional enzymes in fourth-instar and pupal-stage *Bradysia odoriphaga*. (A) The relative gene expression level (log_2_FC) in DEG analysis. (B) The relative activity of enzymes (log_2_R). R indicates the ratio of enzymes activity at different stages.

At the basis of RT-PCR and qRT-PCR validation, activity assay of enzymes was carried out to validate gene expression profiles in protein expression level. The compared results between gene expression profiles and relative activity of enzymes were showed in Fig 8. Except for *glutathione S-transferase 1-like* and *pectate lyase*, the gene expression profiles of the other eight genes (80%) were all consistent with the relative activity of relative activity of enzymes (Fig 8A and 8B).

## Discussion

To discover and annotate the gene involved in important physiological and biochemical process, *de novo* assembly and the analysis of differential expression genes had provided an effective way for the species without reference genome or sequence [31]. Therefore, under the condition of no reference genome of *B*. *odoriphaga*, 47,578 unigenes (≥125bp) were generated from the third-instar, fourth-instar, and pupal *B*. *odoriphaga* through Illumina sequencing and combined *de novo* assembly. The results were similar to those from the study by Chen et al. [21], in which 16,829 unigenes were assembled by *de novo* sequencing in the single larval transcriptome of *B*. *odoriphaga*, namely about one-third of the unigene numbers in the three developmental stages. However, the number of unigenes (21,095) annotated in the NR protein database in all the three developmental stages was not more than twice the unigenes (12,024) in the single larval transcriptome. The same conclusion was also found in the GO annotation (10,023 and 7,903); this was because many unigenes in the third-instar, fourth-instar, and pupal *B*. *odoriphaga* had the same function. Moreover, we found that 9,361 unigenes of *B*. *odoriphaga* matched homologous genes in Diptera insects, such as *A*. *aegypti* sequences followed by *C*. *quinquefasciatus*, *C*. *capitata* and *A*. *gambiae* (Fig 1). Now, genomic research involves many insects, for instance, Hymenoptera [32], Lepidoptera [33,34] and Coleoptera insects [35]. Regarding Diptera, 15,419 coding genes were analyzed in the genome of *A*. *aegypti* [22]. 18,883 genes were discovered in genome of *C*. *quinquefasciatus* [23]. The unigenes of *B*. *odoriphaga* from transcriptome sequencing could provide abundant genetic information at the genomic level for *B*. *odoriphaga* and other Diptera insects.

Through qRT-PCR analysis for 45 genes selected from 485 genes expressed differently among third-instar, fourth-instar, and pupal *B*. *odoriphaga*, it was found that the majority of genes were up-regulated in fourth-instar and down-regulated in pupal stages compared to the third-instar stage (S5 Table). Example of these include ecdysteroid regulated-like protein, metabolism-related genes (*lipase*, *delta-5 desaturase*, *acidic endochitinase*, *pectate lyase*, *lysosomal alpha-mannosidase*, *regucalcin*, etc.), and defense response related genes (*glutathione S-transferase*, *carboxylesterase*, *defensin*, etc.). These conclusions were related to physiological characteristics at each stage of the *B*. *odoriphaga* life cycle. During the life history of *B*. *odoriphaga*, the metabolic activity of insects reaches to the highest point in the last larval stage, which is convenient for finishing every physiological function of larvae [36]. After ecdysis in the fourth-instar stage, *B*. *odoriphaga* enters the pupal stage, in which insects began to store fat and energy for overwintering through reductions in metabolic activities [37,38]. With the exception of the 45 genes expressed differently among third-instar, fourth-instar, and pupal *B*. *odoriphaga*, the DEGs related to the developmental process were selected also from the comparisons of third-instar and fourth-instar larvae, third-instar larvae and pupae, and fourth-instar larvae and pupae. Twenty closely related DEGs of development were selected according to the standard (log_2_ FC≥2) (Table 3 and S3 Table). Many researchers also have discovered genes related to the developmental process through transcriptome sequencing [39–44]. In the horned beetle *Onthophagus taurus*, 8% of variable genes were annotated as involved in the developmental process in the GO database and contributed to horn formation and plasticity in horned beetle development [45]. The genes *ecdysone receptor isoform A* of *O*. *taurus* and *ecdysone-induced protein 63E isoform N* (c26961) of *B*. *odoriphaga* discovered in this study were both related to embryonic development. He *et al*. [46] discovered more than100 unigenes involved in gamete generation, ovarian follicle cell development and mating behavior of *Phyllotreta striolata*. The genes *dynamin* and *glucose dehydrogenase* in *P*. *striolata* were also found in *B*. *odoriphaga* (c4206 and c20905). Therefore, the discovery of similar genes in different species demonstrates the conservative functions of these genes.

Moreover, the expression levels of genes involved in the open tracheal system, heart, and stem cell development were stable in the third-instar and fourth-instar in *B*. *odoriphaga*. During the entire developmental phase of *B*. *odoriphaga*, the third-instar insects had a full-grown circulatory system and a digestive system, and the mostly damaged chives [5], which explains the fact that the related genes, such as (*GK10804* [c24932]), expressed stably during the larval stage. In the pupal stage, the genes involved in the reproductive process, reproductive organ development, and PCD were all up-regulated, and those genes in the circulatory and digestive systems were down-regulated compared to larval-stage *B*. *odoriphaga* (Table 3 and S3 Table). It was related to the phenomenon in which adult *B*. *odoriphaga* took mating and laying eggs instead of feeding behavior completely [10]. Therefore, the organs not related to reproduction, including salivary gland and other digestive organs, also entered into PCD [47–49]. The gene *caspase Nc-like* (c33601) was annotated in the KEGG apoptosis pathway (Ko04210). In the PCD process, production of lysosome organelleS was the important process. Genes *ecdysteroid regulated-like protein* (c17315) and *lysosomal α-mannosidase* (c30916) were annotated in the KEGG lysosome pathway (Ko04142). Therefore, the research of the KEGG pathway related to PCD could provide more regulated information for explaining the developmental mechanism from larval to pupal stages of *B*. *odoriphaga*. However, this will still need further verification through RNA interference (RNAi) technique in the next step. If the function of the key genes related to the developmental process can be demonstraed by RNAi in the next step, the aim of control of *B*. *odoriphaga* can be reached through silencing the key genes.

In our study, although the functions and the related signal transduction pathways of many unigenes have not been discovered, the information from transcriptome and DEG analysis could also provide a genetic basis for researching the developmental mechanism.

## Supporting Information

S1 Fig Distribution of all unigenes of *Bradysia odoriphaga* at three developmental stages. (TIF)

S2 Fig Location distribution of mapped reads in mRNA. Horizontal axis indicates the location of mRNA after dividing the mRNA into100 sections. Vertical axis indicates the ratio of mapped reads in the corresponding location of mRNA. The more even distribution of mapped reads in mRNA means a higher randomness of mRNA fragmentation.
(TIF)

S3 Fig Simulated diagram of saturation test of sequencing data. Horizontal axis indicates the number of reads (106) after dividing the mapped reads into100 sections. Vertical axis indicates the number of detected unigenes (fragments per kilobase of transcript per million mapped reads [FPKM] ≥ 0.1).
(TIF)

S4 Fig Frequency of occurrence of annotated *Bradysia odoriphaga* unigenes in the Eukaryotic Orthologous Groups (KOG) data library. (TIF)

S5 Fig Frequency of occurrence of annotated *Bradysia odoriphaga* unigenes in the Clusters of Orthologous Groups (COG) data library. (TIF)

S6 Fig Correlation plot diagram of expression levels for unigenes of two biological replicates. Horizontal axis and vertical axis indicate the value calculated according to log(fragments per kilobase of transcript per million mapped reads [FPKM] + 1) of two biological replicates of *Bradysia odoriphaga* in each developmental stage (3rd: Third-instar insects; 4th: Fourth-instar insects; Pupa: Pupal insects).
(TIF)

S7 Fig Numbers of 45 differentially expressed genes (DEGs) together in each comparison analyzed by quantitative real-time polymerase chain reaction (qRT-PCR). A: *β*-actin was a reference gene. B: *β*-tubulin was a reference gene. Three comparisons of third-instar and fourth-instar *Bradysia odoriphaga* (3rd vs 4th), third-instar and pupal insects (3rd vs Pupa), and fourth-instar and pupal insects (4th vs Pupa) are shown.
(TIF)

S8 Fig PCR amplification analysis of 45 differentially expressed genes (DEGs) in *Bradysia odoriphaga* at three stages. M: DNA marker (D2000); 1: Third-instar larvae; 2: Forth-instar larvae; 3: Pupa.
(TIF)

S1 Table Primer sequence and fragment length of genes for RT-PCR. (PDF)

S2 Table Primer sequence of genes for qRT-PCR. (PDF)

S3 Table Regulated and annotated information of differentially expressed genes (DEGs) related to *Bradysia odoriphaga* development. (PDF)

S4 Table COG, KOG, and KEGG annotations of differentially expressed genes (DEGs) related to *Bradysia odoriphaga* development. (PDF)

S5 Table Subset of 46 differentially expressed genes (DEGs) selected in this study. (PDF)
