# Supplementary figures and images for: Transcriptome Analysis and Discovery of Genes Relevant to Development in Bradysia odoriphaga at Three Developmental Stages
Source: PLoS One. 2016 Feb 18;11(2):e0146812. doi: 10.1371/journal.pone.0146812 (PMC4759360; doi:10.1371/journal.pone.0146812)

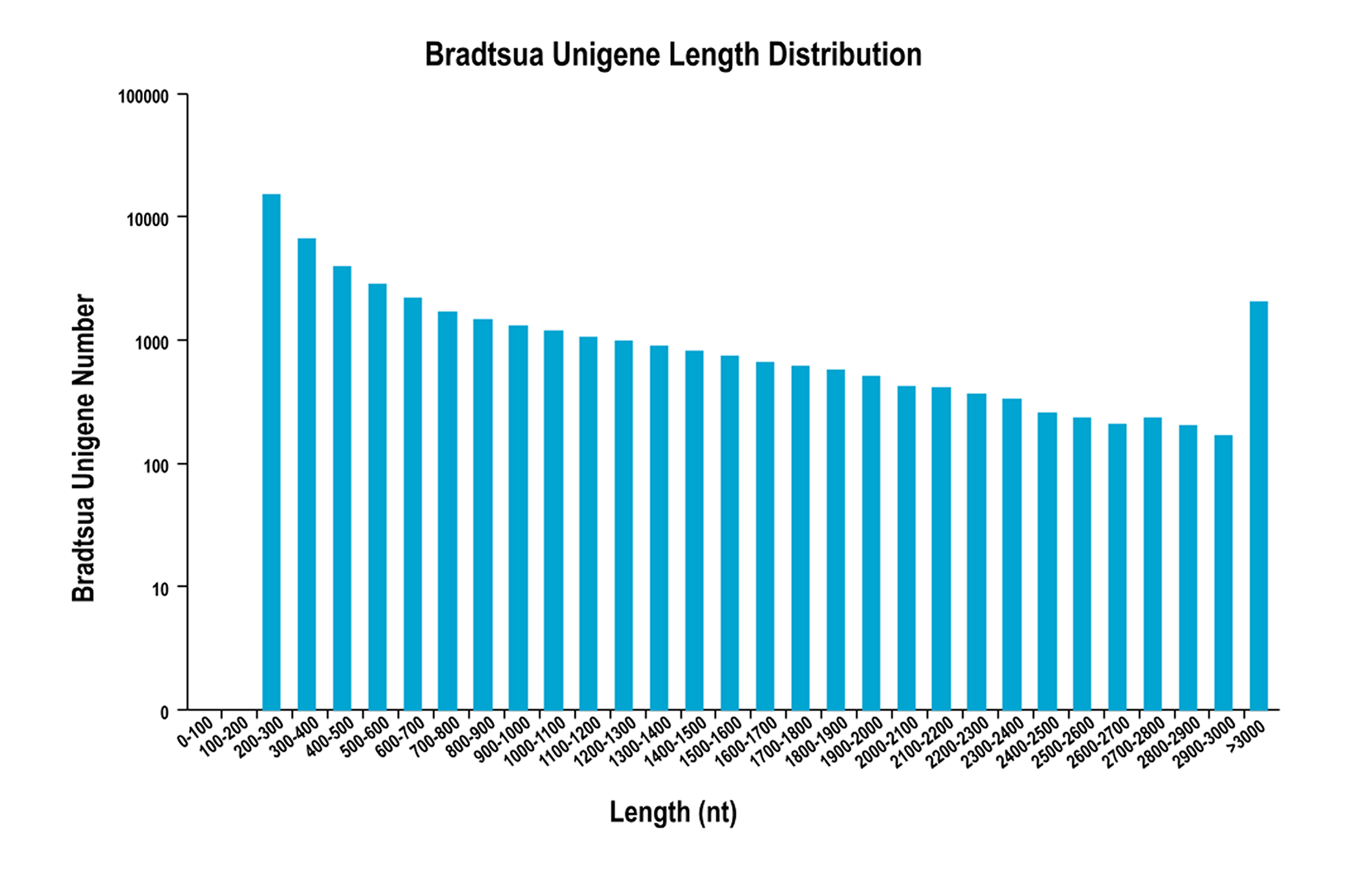

Supplement: S1 Fig — (TIF) [file pone.0146812.s001.tif]

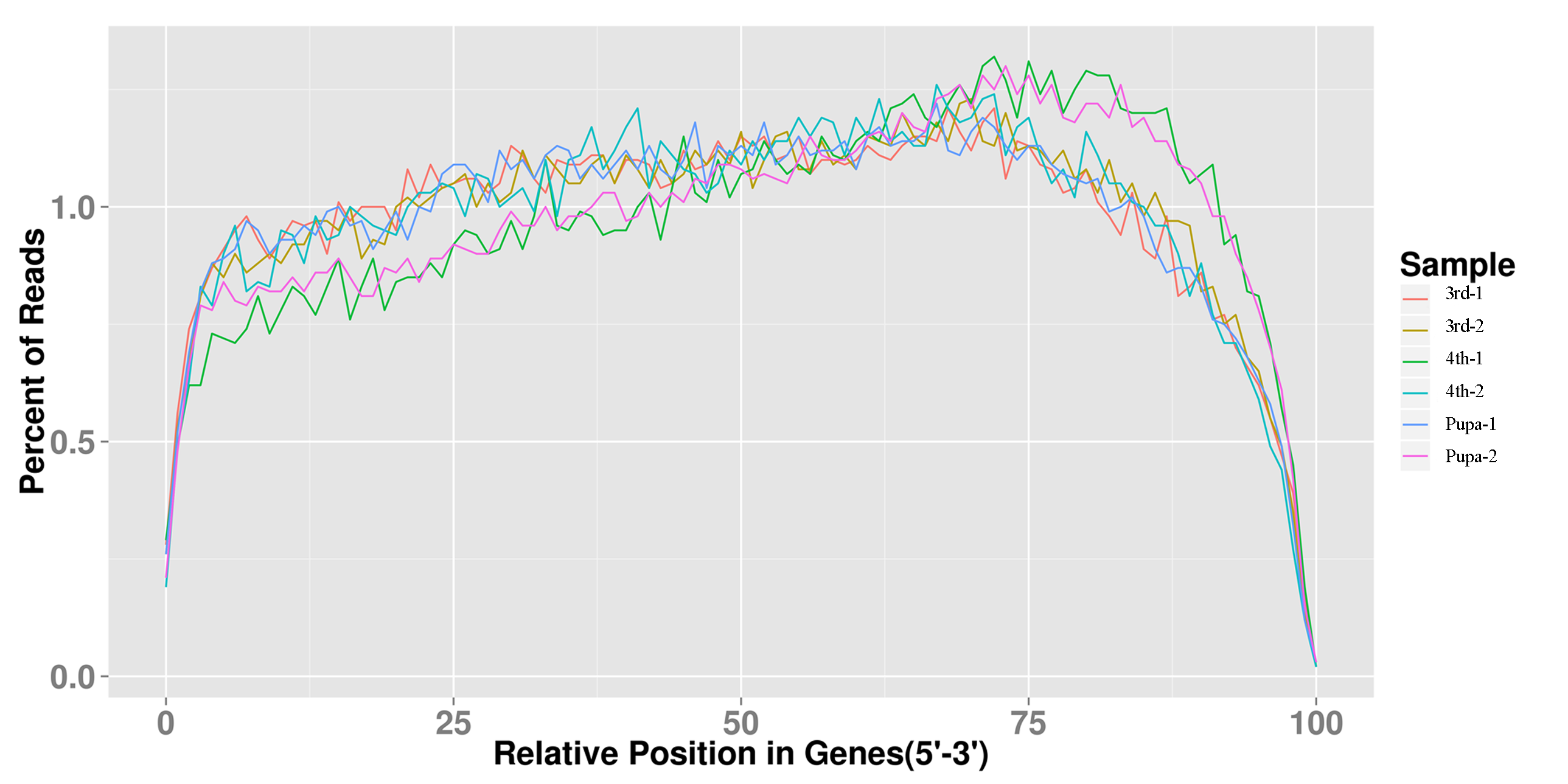

Supplement: S2 Fig — Horizontal axis indicates the location of mRNA after dividing the mRNA into100 sections. Vertical axis indicates the ratio of mapped reads in the corresponding location of mRNA. The more even distribution of mapped reads in mRNA means a higher randomness of mRNA fragmentation. (TIF) [file pone.0146812.s002.tif]

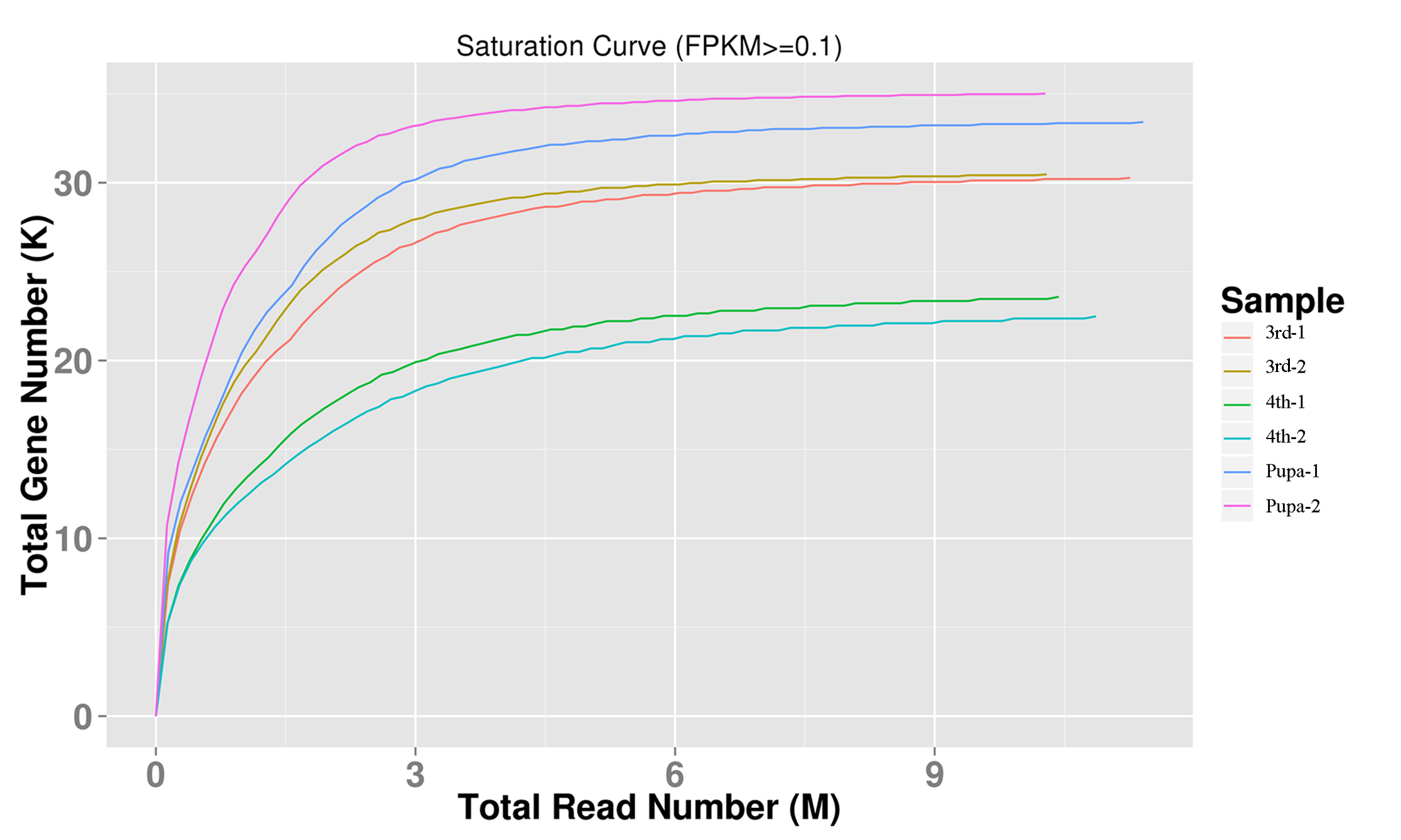

Supplement: S3 Fig — Horizontal axis indicates the number of reads (106) after dividing the mapped reads into100 sections. Vertical axis indicates the number of detected unigenes (fragments per kilobase of transcript per million mapped reads [FPKM] ≥ 0.1). (TIF) [file pone.0146812.s003.tif]

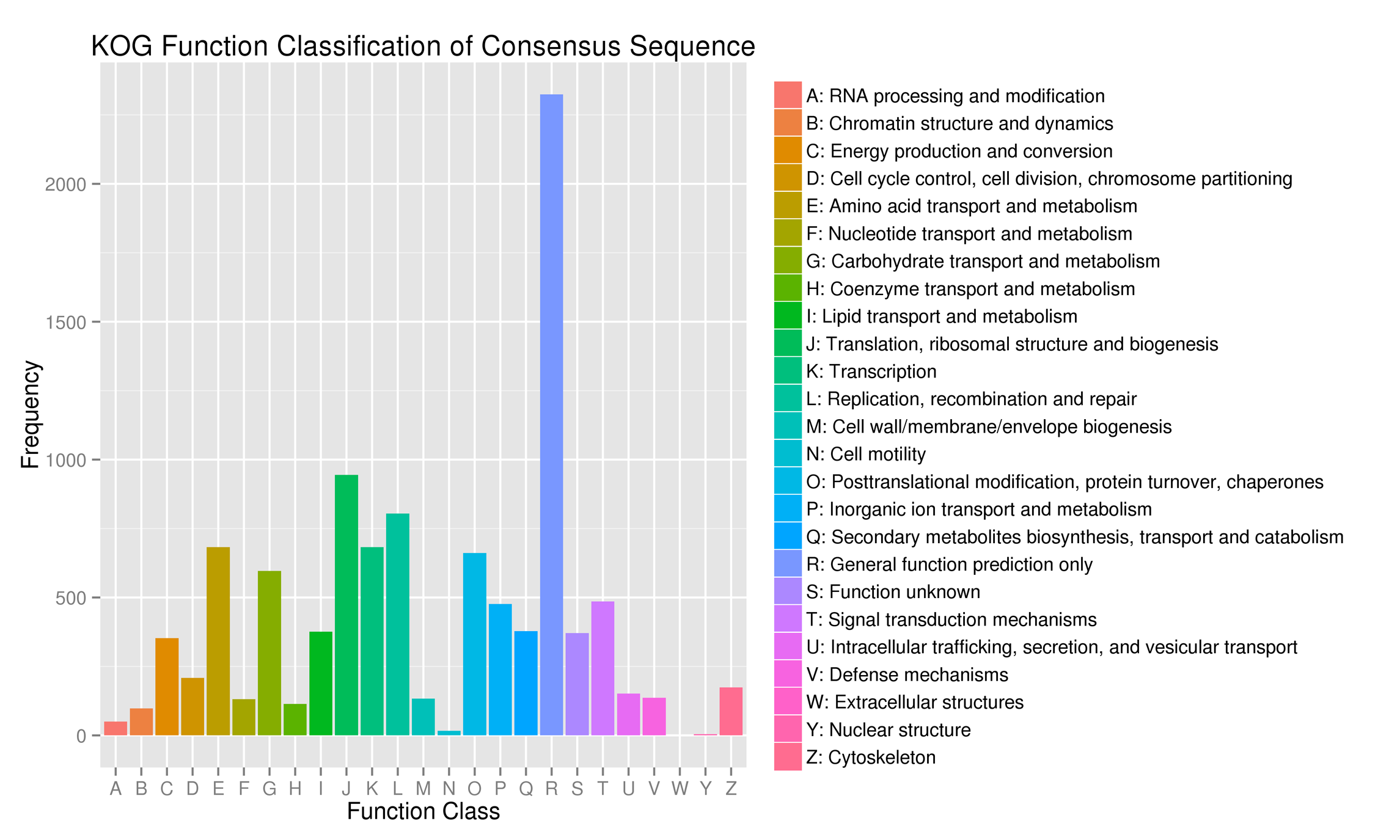

Supplement: S4 Fig — (TIF) [file pone.0146812.s004.tif]

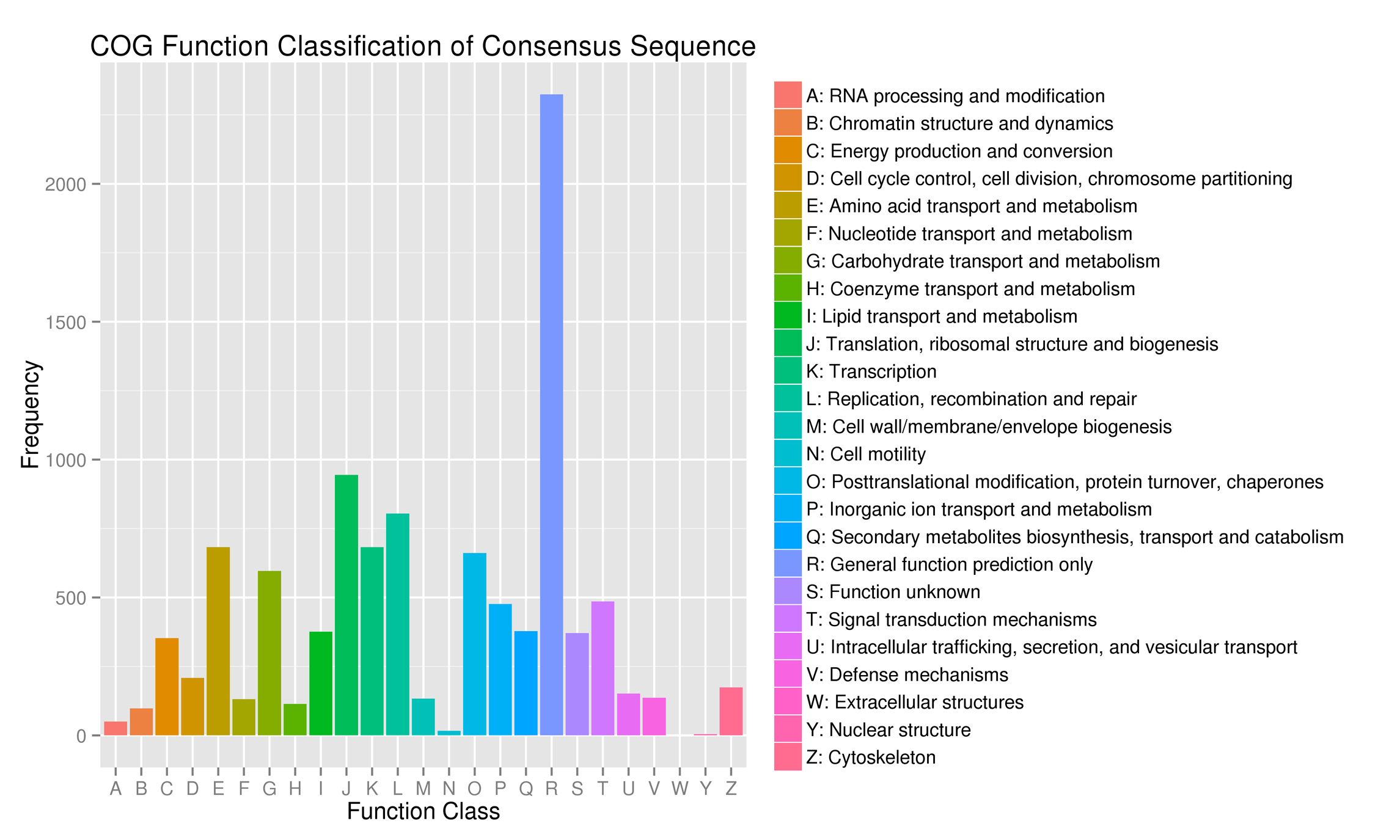

Supplement: S5 Fig — (TIF) [file pone.0146812.s005.tif]

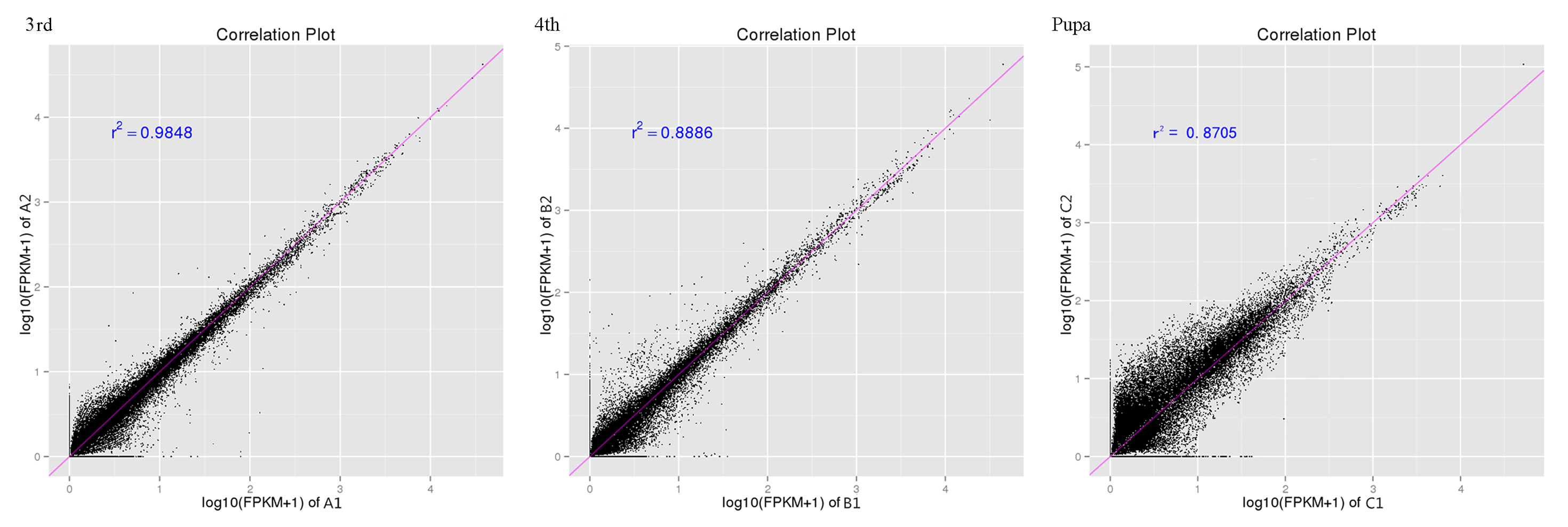

Supplement: S6 Fig — Horizontal axis and vertical axis indicate the value calculated according to log(fragments per kilobase of transcript per million mapped reads [FPKM] + 1) of two biological replicates of Bradysia odoriphaga in each developmental stage (3rd: Third-instar insects; 4th: Fourth-instar insects; Pupa: Pupal insects). (TIF) [file pone.0146812.s006.tif]

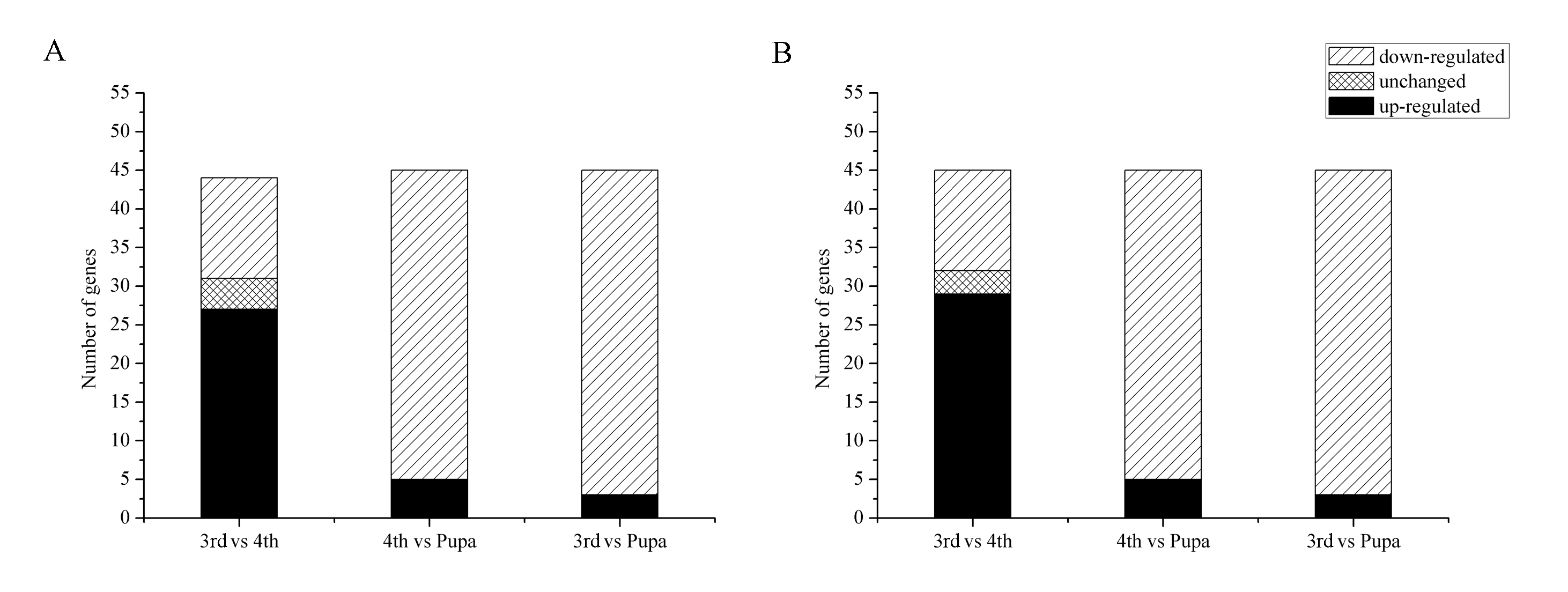

Supplement: S7 Fig — A: β-actin was a reference gene. B: β-tubulin was a reference gene. Three comparisons of third-instar and fourth-instar Bradysia odoriphaga (3rd vs 4th), third-instar and pupal insects (3rd vs Pupa), and fourth-instar and pupal insects (4th vs Pupa) are shown. (TIF) [file pone.0146812.s007.tif]

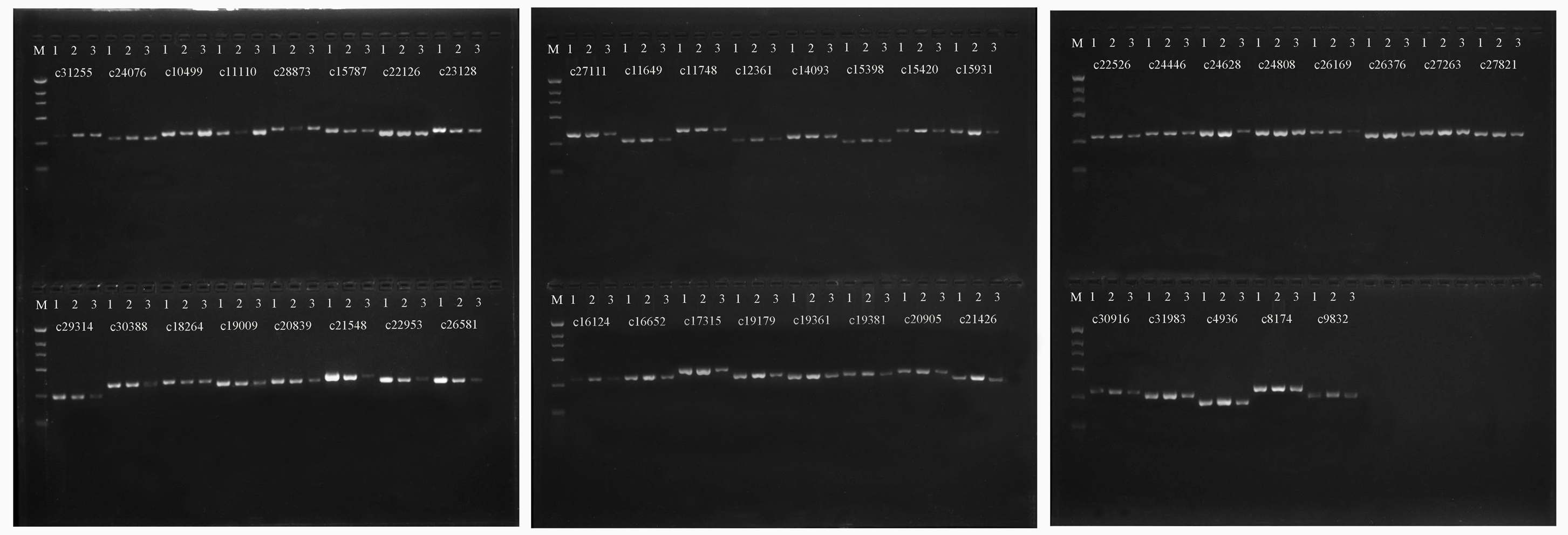

Supplement: S8 Fig — M: DNA marker (D2000); 1: Third-instar larvae; 2: Forth-instar larvae; 3: Pupa. (TIF) [file pone.0146812.s008.tif]
