# Supplementary material for: Transcriptome Analysis and Discovery of Genes Relevant to Development in Bradysia odoriphaga at Three Developmental Stages
Source: PLoS One. 2016 Feb 18;11(2):e0146812. doi: 10.1371/journal.pone.0146812 (PMC4759360; doi:10.1371/journal.pone.0146812)
Supplement: S1 Table — (PDF) [file pone.0146812.s009.pdf]

Table S1 Primer sequence and fragment length of genes for RT-PCR

Genes		Primer sequence (5'- 3')	Fragment length (bp)
c24076	F:	AACCCTCGTTGCTGAGACTG	270
	R:	AGCCATTCCCTTGTTGACTG	
c31255	F:	AAATGCGAATCAATGCACAG	304
	R:	TCCACCTCCACTGCCTTTAC	
c15787	F:	ACCAATGCCAACGACAAAT	300
	R:	ACAAGCCGAGTGGGTGTAAT	
c22126	F:	AATGCTTGTGGCTCTGTCGT	284
	R:	GTGGCTGGGTGGAATGAAT	
c23128	F:	CATCCTGCTGCTCTTTCACA	311
	R:	ACTGTTTTCGGTCCCTCTCC	
c29314	F:	GATTGGGTGTGGATTGTTCC	298
	R:	CGCATTGGTTCGTGTTCTTA	
c30388	F:	TACGACAATGCTGCCGATAA	323
	R:	GCGACTGACGATGATAAAAGG	
c10499	F:	TTCACCAGGCAACAACAGAC	304
	R:	ACCAAAGACAAGCGGAAAAG	
c11110	F:	CTCCTCAAATCCCACTCCAG	297
	R:	AGCCAGTTCCTCCAATGCT	
c28873	F:	ATCTCAGCGAAGCAACCATT	328
	R:	TTCATACCATCCCCAAAGGA	
c11649	F:	TATTCACACCAACGGTCCAA	257
	R:	TATCACCACCCATCCATTCA	
c11748	F:	GGGAACGGACAAGAATACCA	336
	R:	CCCTCACGAACCAACAAAAT	
c12361	F:	AGCTGCGGAGATGGTTCA	246
	R:	TTGGTTATTGACAGCGTGGA	
c14093	F:	CTGCGAAACGGTAATGAACC	265
	R:	GGGTGGACAGGAACTCACTC	
c15398	F:	TTTGTATGGATGTGGTGACAGA	227
	R:	GTCCCGAGCGATTCTTGA	
c15420	F:	TCCGCTTGTGATTGTTGAAG	303
	R:	AGTCGCGCACGTAAAACATT	
c15931	F:	TGGAGCAAGCGTAAAACTCA	298
	R:	TGAACCGTCTAACCAAACTCC	
c16124	F:	CCAGGCAACTTTATCGCAGT	290
	R:	TGTCCAACAAGGTCATTTCG	
c16652	F:	AGGTGGACGAGGGTGTAGAG	299
	R:	TTTGTTGGTGTGACTGTAGGC	
c17315	F:	GCATCATCTCGGACTGTGAA	357
	R:	CGCATCTTGTGTTTCTGGTC	

c18264	F:<br>R:	CGGTTTTCCGTCTATCTCTCC<br>ATTATCTGTGAAGCAGCAATGG	333
c19009	F:<br>R:	CTATGATTGTGCCGATGGTG<br>TGGAAGAAGCCAGCAGTTTAT	308
c19179	F:<br>R:	CGCTGGCTGTTGTTAGGAG<br>CGCACTTGGAAACGATACTG	296
c19361	F:<br>R:	TCCAGCCCTTCGTTACTACAA<br>CCACCGAGCAATCTCTTAGC	285
c19381	F:<br>R:	TCTGGTGTCGTTCACAAAGG<br>ATGGCAGTCCAAGACGCTAC	296
c20839	F:<br>R:	ATGGAACACTGGGAAAATCG<br>TCGGACATAGCAAAAGAATGC	316
c20905	F:<br>R:	ATGGTCAACGACACAGCACT<br>GGAAAAATGGAACCGTCAAA	330
c21426	F:<br>R:	GGAAAATCCGTTGTGTTCGT<br>TAACTCCGTCAGCCCAAACT	287
c21548	F:<br>R:	CCAAGCGTATTCTCATCCACT<br>GGGCACAGTTCAGTTTCTCTC	349
c22526	F:<br>R:	TGTCGGAAATAGATGATGTTCG<br>TCCCAGATTCGTTTTGAAGC	292
c22953	F:<br>R:	ACTCAAACCGCACTTGGCTA<br>ATTGCTTACCGTTCCATTCG	323
c24446	F:<br>R:	TGCGTTTCTGGTGTCTGATT<br>TCATTCGTCTGCTTTCCTGA	308
c24628	F:<br>R:	GACTGGAAGCAATCCTCGTC<br>TGTGCGATACTGAGCGTTTC	313
c24808	F:<br>R:	AGGACTGTATCCGCCGTAAT<br>GAATGGTTGCCGCTTATTTT	318
c26169	F:<br>R:	CAGCCGATAAAAATCCCGTA<br>ACAAGGGCAAGTCAATGTCC	305
c26376	F:<br>R:	TGGCGTTGATGCTAACTGAA<br>AAAGCGGGTAGTGTGACTGG	283
c26581	F:<br>R:	CAGGAAACGAGACGGATTGT<br>ATGCCCCGAGTAAGGCTATC	317
c27111	F:<br>R:	CGTCAGCATCACAAATCCAT<br>GCAGTGGCAAAAGAGGAAAA	313
c27263	F:<br>R:	GATTTGATGTTCGCCGTTG<br>CATTTGGTGGTGTTGGATTTC	295
c27821	F:<br>R:	ATACCAATGCGGAAGAGACG<br>CCAGTCAACGGCAAAGAAAT	272
c30916	F:<br>R:	TTCAGCCACAGCGACTACAC<br>TCTCCGACCACTCACAAACA	290
c31983	F:<br>R:	GACCAAATGTCTCCATCCGTA<br>GGACCCTCAAGCAAACACA	267

c4936	F:	GTCTTGTTGCGGGTCAATCT	218
	R:	GTCACACCACACTTGGAACG	
c8174	F:	TTGTTCCGCACCTTCCAC	305
	R:	GTTTTCAATGGGCTTCGGTA	
c9832	F:	TTCTCTGCTTTTCCCTCAGC	246
	R:	AAGTGGACCGTTTCTGTGC	
