# Supplementary material for: Transcriptome Analysis and Discovery of Genes Relevant to Development in Bradysia odoriphaga at Three Developmental Stages
Source: PLoS One. 2016 Feb 18;11(2):e0146812. doi: 10.1371/journal.pone.0146812 (PMC4759360; doi:10.1371/journal.pone.0146812)
Supplement: S2 Table — (PDF) [file pone.0146812.s010.pdf]

Table S2. Primer sequence of genes for qRT-PCR

Genes		Primer sequence (5'- 3')
β -actin	F:	CTGTCCACCTTCCAAACGAT
	R:	AACACAGAAGAAACGACACCA
β -tubulin	F:	GAAGAAATGAAGACGTGGAA
	R:	ATGACGATGTCGGGAGTG
c24076	F:	CGTCTAATGATGTGTTCGTTGC
	R:	TGTTGCTTCAGGGAAATACTCTAAC
c31255	F:	GCAGGCGTAAATCAGAGTGG
	R:	TCAGGGACAATCCAGGTAAGA
c15787	F:	CCAGGTCGTTTGTATTGTAGCA
	R:	ATTGCGTTCTTGGTGGATG
c22126	F:	ACTTCGTTCAGCAGGTCACA
	R:	CGATGGATTGTTCGGTCATA
c23128	F:	AGGGTATGGCGAAGTGATTG
	R:	GTATGGGACCGCTGAGAGTG
c29314	F:	TCACATCACGACTGCTTCG
	R:	TGCGGAAAGACTGTAACTGC
c30388	F:	CATTTCACTTTGGCTGCGTA
	R:	AAACGGCTTTCAACTCTCCA
c10499	F:	TTTATCGGGTGTCGGAGATG
	R:	GTCTGTTGTTGCCTGGTGAA
c11110	F:	CAACCCAGCAAACCAGAAGT
	R:	GATTCCTCCACCGATTCCTC
c28873	F:	TCACGAACTTGGCGTAACTG
	R:	GTCATTTGGACGAACAGAAACA
c11649	F:	CTTGTTCAGCGTGCGTAGTC
	R:	CTCCAATACCGAGTCCGATG
c11748	F:	CGACACTTCCGCTTAGTCAA
	R:	ACACCACCCTCCACCATTT
c12361	F:	GGCCATCATATCTGGTCTTAATG
	R:	GCGTGGAAGAGAGCTTCAAT
c14093	F:	CATCGGGTTCGGTGAGAAT
	R:	TGTCTTCGTCGCTGTTGAAA
c15398	F:	TTTAGTTGTGTCCTTGCACCA
	R:	GCTTCAAACCGAAATCAACA
c15420	F:	CTTCCGCTTGTGATTGTTGA
	R:	TGTGCTTCTATTATTGGCTGCT
c15931	F:	CGAATGGACAACCGTAATCA
	R:	TGAACCGTCTAACCAAACTCC
c16124	F:	CGAAACGGCTCAAAGAGAAT
	R:	TGAACATTACCGCACTCCTG
c16652	F:	TATGTGCCTGTGTGCGAATG
	R:	TCTTTCTGGCGTTGGCTTAT
c17315	F:	CGGACTGTGAATCTGACTGG
	R:	CACTATGTTGACCGCAGGAT
c18264	F:	CATGGCAGTGAATCGGAATA

	R:	AGCAATGGGAATCGAAATGA
c19009	F:	GATTATGTGGGAATGGTGTCG
	R:	TGACGAATACTGAATGGAATGC
c19179	F:	TGAAGGAAGAATCCCGAATAG
	R:	CTAAACGCACTTGGAAACGA
c19361	F:	GCCCAGAAAGCAATAGTCAAA
	R:	AGCCACCAAGTCAACAAGGA
c19381	F:	CCGATGCTGGATGTAGTTGA
	R:	CTTGGTGTGGTGGATGTTTG
c20839	F:	TTGTCACCATTATCCCACCA
	R:	GTCTCCTACATTCGGGTCCA
c20905	F:	GAAGTGATTTGATGGTTGGACA
	R:	CCCGATTTGTTTATGAGAAGG
c21426	F:	GCTCGTTATGGGCAACCA
	R:	GTAGCACCTTTCAGCCAACA
c21548	F:	CCGCCATTACAACTTCGTTT
	R:	GGATGAGAATACGCTTGGTG
c22526	F:	GATTTCCACTGACCGAATGG
	R:	TCAAACCGTCGTATGTCCAC
c22953	F:	GGCGGCATCTATTTCGTG
	R:	ACACTCAAGGGCTGGTATCG
c24446	F:	AAATGGTTTCGGTGACTTCG
	R:	GAATAAGGATAGTGCTGCGTGA
c24628	F:	ACAGCGATTCTTTCCCAGAG
	R:	TGTGCGATACTGAGCGTTTC
c24808	F:	TCGCAGCATTGATTACCAGA
	R:	GAACGGTGGAAGGAAGTAGC
c26169	F:	GGACATTGACTTGCCCTTGT
	R:	GGAGTGGGTTGTGTTTGGTC
c26376	F:	ATTTCTACCACGCTGCCACT
	R:	CGCTTCTACTGCTGCCTACC
c26581	F:	CAGGAAACGAGACGGATTGT
	R:	ACTGGGCGAAACTGCTTAGT
c27111	F:	GCCGACCCACCTACGATAA
	R:	ATGGATTTGTGATGCTGACG
c27263	F:	ACAATGCCAATCCACACATC
	R:	CAACGGCGAACATCAAATC
c27821	F:	AGTCGGAGAGTGTGGACGAG
	R:	TCCCAGAGAATGACCCAGAA
c30916	F:	GAAGCAGGAACCCACTACCA
	R:	GATTTGCCAGCCCACTTTC
c31983	F:	CCCTGTTGTATGATTCGGAGA
	R:	ACGGATGGAGACATTTGGTC
c4936	F:	CACAAACGGCTGACAACG
	R:	CGCCTCAACTTCCTTACTTTCA
c8174	F:	TCCCGAATGTGGTATGTCTC
	R:	CGGAAGATGTGCGAGTAACA
c9832	F:	CGCATCATTTCTTGGACATC

R: AAAGTGGACCGTTTCTGTGC
