# Supplementary material for: Transcriptome Analysis and Discovery of Genes Relevant to Development in Bradysia odoriphaga at Three Developmental Stages
Source: PLoS One. 2016 Feb 18;11(2):e0146812. doi: 10.1371/journal.pone.0146812 (PMC4759360; doi:10.1371/journal.pone.0146812)
Supplement: S3 Table — (PDF) [file pone.0146812.s011.pdf]

Table S3. Regulated and annotated information of differentially expressed genes (DEGs) related to *Bradysia odoriphaga* development

Unigene ID	3th vs 4th		4th vs Pupa		3th vs Pupa	
	log2FC	Regulated	log2FC	Regulated	log2FC	Regulated
c4843	-5.17	down	--	--	-5.97	down
c23897	-7.09	down	--	--	-6.85	down
c33601	3.39	up	--	--	--	
c10244	--	--	2.86	up	2.55	up
c20752	--	--	4.11	up	4.64	up
c23801	--	--	3.58	up	3.46	up
c26961	--	--	2.26	up	2.90	up
c27681	--	--	3.31	up	3.54	up
c28314	--	--	2.65	up	3.52	up
c28746	--	--	3.09	up	3.07	up
c30336	--	--	3.46	up	3.43	up
c30501	--	--	3.78	up	4.65	up
c30850	--	--	2.52	up	2.54	up
c31327	--	--	2.64	up	3.05	up
c4206	--	--	--	--	-4.35	down
c15127	--	--	--	--	2.18	up
c22888	--	--	--	--	4.02	up
c22990	--	--	--	--	2.50	up
c24932	--	--	--	--	-3.75	down
c29289	--	--	--	--	3.49	up

3rd vs 4th: the comparison of third-instar and fourth-instar *B. odoriphaga*; 4th vs Pupa: the comparison of fourth-instar and pupal insects; 3rd vs Pupa: the comparison of third-instar and pupal insects; The "up" or "down" indicates the gene of latter one up-regulated or down-regulated compared with the fomer one. For example, "down"in "3rd vs 4th" indicates down-regulated in fourth-instar compared with third-instar stage.
