# Supplementary material for: Transcriptome Analysis and Discovery of Genes Relevant to Development in Bradysia odoriphaga at Three Developmental Stages
Source: PLoS One. 2016 Feb 18;11(2):e0146812. doi: 10.1371/journal.pone.0146812 (PMC4759360; doi:10.1371/journal.pone.0146812)
Supplement: S4 Table — (PDF) [file pone.0146812.s012.pdf]

Table S4. COG, KOG and KEGG annotation of differentially expressed genes (DEGs) related to *Bradysia odoriphaga* development

Unigene ID	COG annotation	KOG annotation	KEGG annotation
c4843	Transcription	Transcription	—
c23897	General function prediction only	—	—
c33601	—	Cell cycle control, cell division, chromosome partitioning	Apoptosis (Ko04210)
c20752	—	General function prediction only	—
c23801	—	Signal transduction mechanisms	MAPK signaling pathway - fly (ko04013)
c26961	—	General function prediction only	—
c31327	—	Cell cycle control, cell division, chromosome partitioning	TGF-beta signaling pathway (ko04350)
c4206	—	—	Endocytosis (ko04144)
c29289	—	Replication, recombination and repair	—
c11110	Carbohydrate transport and metabolism	—	—
c29314	—	Energy production and conversion	Arginine and proline metabolism (ko00330)
c16124	—	General function prediction only	—
c20905	Amino acid transport and metabolism	General function prediction only	—
