# Supplementary material for: Transcriptome Analysis and Discovery of Genes Relevant to Development in Bradysia odoriphaga at Three Developmental Stages
Source: PLoS One. 2016 Feb 18;11(2):e0146812. doi: 10.1371/journal.pone.0146812 (PMC4759360; doi:10.1371/journal.pone.0146812)
Supplement: S5 Table — (PDF) [file pone.0146812.s013.pdf]

Table S5. Subset of 45 differentially expressed genes (DEGs) selected in this study

Regulated type	Unigene ID	3rd vs 4th		4th vs Pupa		3rd vs Pupa		NR annotation
		log2FC	Regulated	log2FC	Regulated	log2FC	Regulated	
Ⅰ	c24076	2.07	up	4.07	up	6.36	up	no result
	c31255	1.72	up	3.22	up	5.16	up	fatty acid synthase
Ⅱ	c15787	-1.14	down	-5.16	down	-6.08	down	Rer1 protein
	c22126	-3.21	down	-8.78	down	-11.79	down	multiple inositol polyphosphate phosphatase
	c23128	-1.09	down	-2.65	down	-3.53	down	reverse transcriptase-like protein
	c29314	-1.21	down	-3.58	down	-4.56	down	Nitric oxide synthase (NOS)
	c30388	-3.77	down	-3.70	down	-7.26	down	no result
Ⅲ	c10499	-2.65	down	5.33	up	2.91	up	histone H2B
	c11110	-6.56	down	9.66	up	3.33	up	probable H/ACA ribonucleoprotein complex subunit 1-like
	c28873	-1.14	down	8.58	up	6.34	up	cytochrome P450, putative
Ⅳ	c11649	1.58	up	-13.98	down	-12.15	down	lipase
	c11748	1.14	up	-4.64	down	-3.27	down	ribonuclease UK114-like
	c12361	3.68	up	-10.42	down	-6.52	down	no result
	c14093	2.88	up	-14.70	down	-11.56	down	beta-1,4-endoglucanase
	c15398	1.22	up	-4.91	down	-3.47	down	cytochrome P450 2U1-like
	c15420	1.05	up	-10.48	down	-9.19	down	thioredoxin domain-containing protein 12-like isoform X2
	c15931	2.09	up	-13.62	down	-11.29	down	defensin
	c16124	1.15	up	-14.55	down	-13.16	down	acidic endochitinase SP2-like
	c16652	2.49	up	-6.11	down	-3.41	down	putative trypsin Inhibitor like cysteine rich domain protein
	c17315	2.54	up	-10.65	down	-7.91	down	ecdysteroid regulated-like protein

c18264	1.95	up	-5.51	down	-3.33	down	cytochrome P450 CYP4D3v2
c19009	1.18	up	-8.33	down	-6.92	down	multiple inositol polyphosphate phosphatase
c19179	1.03	up	-10.15	down	-8.89	down	puff B-2 protein
c19361	1.94	up	-6.52	down	-4.36	down	hexamerin 2 beta
c19381	1.28	up	-8.11	down	-6.61	down	phytanoyl-CoA dioxygenase, peroxisomal-like
c20839	1.1	up	-7.91	down	-6.6	down	alpha-amylase
c20905	2.06	up	-11.06	down	-8.75	down	glucose dehydrogenase
c21426	1.25	up	-11.55	down	-10.05	down	acidic endochitinase SP2-like
c21548	1.74	up	-11.28	down	-9.31	down	similar to metalloproteinase inhibitor 3
c22526	1.23	up	-3.77	down	-2.32	down	delta-5 desaturase
c22953	1.01	up	-9.18	down	-7.92	down	AMP dependent ligase
c24446	1.08	up	-11.87	down	-10.53	down	protease m1 zinc metalloprotease
c24628	1.72	up	-14.10	down	-12.12	down	beta-1,4-endoglucanase
c24808	1.13	up	-11.21	down	-9.84	down	putative oxidase/peroxidase
c26169	1.02	up	-4.08	down	-2.85	down	sialin-like
c26376	1.36	up	-10.44	down	-8.85	down	pectate lyase
c26581	1.29	up	-9.59	down	-8.05	down	peroxidase-like isoform 2
c27111	1.28	up	-6.76	down	-5.26	down	glutathione S-transferase
c27263	1.75	up	-13.51	down	-11.51	down	carboxylesterase
c27821	3.69	up	-7.59	down	-3.68	down	lipase
c30916	1.88	up	-9.40	down	-7.28	down	lysosomal alpha-mannosidase
c31983	1.68	up	-10.69	down	-8.78	down	regucalcin-like
c4936	1.2	up	-4.67	down	-3.27	down	fibrinogen C domain-containing protein 1-like
c8174	1.16	up	-8.75	down	-7.39	down	Lipase family protein
c9832	1.63	up	-8.10	down	-6.24	down	glutathione S-transferase 1-like

3rd vs 4th: the comparison of third-instar and fourth-instar B. odoriphaga; 3rd vs Pupa: the comparison of third-instar and pupal insects; 4th vs Pupa: the comparison of fourth-instar and pupal insects
